# A distributed segmentation strategy developed for three-dimensional leaf trait quantification of tomato plants

**DOI:** 10.3389/fpls.2026.1839309

**Published:** 2026-08-04

**Authors:** Bolai Xin, Yilin Lie, Xiyuan Pan, Mingjie Zhan, Kunming Lv, Jun Li

**Affiliations:** College of Engineering, South China Agricultural University, Guangzhou, China

**Keywords:** deep learning, distributed segmentation, phenotyping, point cloud, tomato plant

## Abstract

Leaf parameters are crucial indicators reflecting the growing status of plants. Monitoring and analysis of leaf parameters significantly contributes to the improvement of crop yield and food quality. This study focused on three tomato plant varieties commonly grown in the Netherlands and proposed a fully automatic pipeline for leaf phenotyping. Three-dimensional (3D) point clouds of target plants were acquired with a specially designed imaging unit naming Maxi-Marvin. A semantic segmentation of plant organs was performed with PointNet++ model. To mitigate point cloud resolution decrement, the down-sampling operation in the baseline model was replaced with a distributed segmentation strategy. Leaf instances were further identified with Density-Based Spatial Clustering of Applications with Noise (DBSCAN), followed by a morphological phenotypic trait quantification based on 3D geometrical analysis. Target phenotypic traits including leaf length, leaf width, and leaf area. The evaluation results indicated that the distributed segmentation strategy achieved the best *F*1 scores of 0.98 with block size set to 30,000. The Mean Average Errors (MAE) of leaf length, leaf width, and leaf area estimation were 2.09 cm, 1.78 cm and 8.98 cm^2^ respectively. The estimation accuracies for leaf length, leaf width, and leaf area were 91.98%, 92.66%, and 89.67%, respectively.

## 1 Introduction

Tomato plants as an important economic crop, are widely cultivated in United States, China, Netherlands and Italy (Cardellicchio et al., 2025). With the rapid development of artificial intelligence and smart agriculture, scientific agronomy and precision tomato management have become increasingly essential for efficient and high-quality production. Plant phenotyping delivers quantitative and timely measurements reflecting plant developmental physiology, enabling data-driven stress mitigation, yield optimization. Among various types of plant organs, leaves are central to photosynthesis and transpiration, making their phenotypic traits strong indicators of plant quality and annual yield. Leaf length, leaf width, and leaf area are key phenotypic traits commonly used to characterize plant growth and development, as leaf size plays a vital role in plant productivity and physiological processes (Abhipray et al., 2022). For example, Cheng et al., 2021 developed a predictive model based on leaf morphology across 18 traditional tomato varieties, indicating that the roundness of plant leaves is positively correlated with Brix values and yields. Cheng et al., 2021 conducted a 14-week field experiment using genetically diverse heirloom tomatoes. Using partial least squares (PLS) regression analysis, they found that leaf morphological traits exhibited significant positive effects on fruit sugar content and yield. Tomato varieties with round leaf morphology, such as Stupice and Glacier, exhibited the highest fruit sugar content and yield.

Traditional plant phenotyping still predominantly relies on human work. However, manual measurements are hindered by high labor intensity, low efficiency, and substantial subjective errors (Mei, 2024), which limits its ability to high-precision and large-scale crop phenotyping. With the rapid advancement of imaging technique, computer vision provides a feasible solution towards high-throughput plant phenotyping. For example, Xiang (2022) proposed a two-dimensional (2D) image segmentation algorithm that utilizes image processing techniques including grayscale conversion, binarization, edge detection, and noise removal to extract shape, texture, and color features of lettuce, enabling accurate representations of lettuce leaf area. Shakoor and Mockler (2022) developed a Raspberry-Pi-based greenhouse camera network in their research. By capturing 2D RGB images from multiple perspectives, they achieved automated 2D phenotyping of crops such as tomatoes and *Arabidopsis thaliana*, extracting key parameters like plant height and leaf inclination angle. Tabb et al. (2022) developed a 2D phenotyping pipeline on mobile phones. Through embedding calibration patterns and utilizing EXchangeable Image Format (EXIF) metadata, their work successfully measured plant morphological parameters including leaf area and plant height. Boogaard et al. (2020) developed a node-detection algorithm for cucumber plants using multi-view imaging and YOLOv3 model. While 2D image processing has proven effective for quantifying a range of plant traits, it remains limited in resolving the three-dimensional (3D) architecture in plants with complex morphologies.

With the rapid development of 3D sensing techniques, point cloud processing offers a feasible solution towards the above challenge. To identify and localize target organs within point cloud data, semantic and instance segmentation of crop point clouds is necessary. Point cloud segmentation methods can be divided into two types: traditional methods with manual feature engineering and fully automatic approaches with deep neural networks. Traditional segmentation firstly extracts discriminative features from the point cloud manually. An auto-thresholding or machine-learning-based classifier was subsequently employed to divide the points into distinct categories. For example, Levashev (2019) initially partitioned the 3D laser data into non-overlapping super-pixels, and segmented the objects by merging these super-pixels according to the following similarity measures: i) distance between the intensity distribution histograms; and ii) distance between the coordinates of points. When compared with conventional segmentation methods that rely exclusively on geometric features, the incorporation of intensity information increased the overall accuracy from 72.3% to 86.7%. Zhao et al. (2021) conducted a technical investigation and evaluation of traditional point cloud clustering methods for panoramic segmentation of lidar data. By developing a hybrid framework integrating semantic segmentation network (Cylinder3D) and clustering algorithm, four representative clustering methods - Euclidean distance clustering, hypervoxel clustering, depth clustering, and scan line running sum clustering - were implemented and evaluated over the Semantic KITTI benchmark. Results demonstrate that the hybrid framework achieves a panoramic quality of 72.3%. The aforementioned segmentation approaches rely heavily on handcrafted geometric features, which fail to capture hierarchical and multi-scale features. As a result, their generalization ability across different plant species and growth conditions is limited, particularly in the presence of organ occlusion, overlapping, motivating the adoption of deep learning-based methods.

Deep-learning-based point cloud segmentation uses Multiple Layer Perceptions (MLPs) and convolutional layers to automatically extract discriminative features of target, followed by fully connected layers to perform regressions between feature space and the class label. As an early milestone, Qi et al. (2017a) proposed the PointNet Vanilla configuration, which introduced shared MLPs and symmetric functions to capture permutation-invariance global features from unordered point sets. To further extract multi-scale local features, Qi et al. (2017b) proposed PointNet++ configuration by introducing set abstraction and feature propagation module. To enhance the representation of local neighboring point features, Thomas et al. (2019) proposed Kernel Point Convolution (KPConv), which defines convolution kernels as a set of learnable points distributed in 3D space, enabling effective modeling of local spatial relationships in irregular point clouds. More recently, transformer-based architectures have been explored to capture long-range dependencies. For example, Tran et al. (2024) proposed PointCT, an end-to-end transformer network for weakly supervised semantic segmentation. By leveraging a center-based attention mechanism, PointCT addresses the challenge of sparse annotations and enhances feature representation through global context modeling.

The advancement of deep-learning-based 3D segmentation has significantly promoted 3D plant phenotyping. For example, Schunck et al. (2021) developed a temporal 3D point cloud dataset for maize and tomato, covering the early vegetative stages and alleviating limitations associated with 2D imaging, such as sensitivity to illumination and viewpoint. Based on this dataset, deep learning models including PointNet and PointCNN were applied to segment plant organs and extract phenotypic traits such as plant height and leaf area index. To quantify structural phenotypic traits, Choi et al. (2024) developed a NeRF-based 3D reconstruction pipeline specifically for tomato crops, enabling accurate extraction of morphological parameters such as plant height, leaf area, and canopy volume from multi-view RGB images. This approach overcomes occlusion challenges inherent in traditional 3D scanning and provides biologically plausible models suitable for growth monitoring and simulation. To quantify structural phenotypic traits, Xin et al. (2023) developed an automated pipeline that combines PointNet++-based semantic segmentation with density-driven point cloud refinement and TreeQSM modeling, enabling quantitative analysis of plant structural traits at multiple scales.

Despite these advances, traditional 3D segmentation networks are generally constrained to a specified number of input points due to the limitations on network capacity and computational capability. Consequently, down sampling is commonly applied to reduce and normalize the number of points, inevitably lowering point cloud resolution and giving rise to loss of fine-grained structural details. A reduction in point cloud resolution adversely impacts the precision of subsequent phenotypic measurements. To overcome the above challenge. Xin et al. (2024) proposed a distributed segmentation strategy. A non-replacement random resampling was employed to divide the original point cloud into multiple subsets. After each subset was segmented independently, the resulting segmentations were merged to produce the final segmentation result. This approach effectively mitigates the resolution loss caused by down sampling, thereby ensuring sufficient surface point coverage for the subsequential Quantitative Structural Modeling (QSM). The overall automated segmentation achieved an accuracy of 0.89, an *F*1 score of 0.71, and a root mean square error (rRMSE) ranging from 4.8% to 64.4%. However, the block partitioning method proposed by Xin et al. (2024) suffers from a notable limitation, as the sampling probability assigned to each point obeys the following conditional probability distribution: $P_{i}=P(p_{i}|p_{1},p_{2},\ldots ,p_{i-1})$, where *P_i_* represents the probability that the *i^th^* sampling result is *p_i_*. This gives rise to variations in local point density among the resulting subsets. The uneven point spatial distribution makes the local density of points a less reliable and discriminative feature, thereby degrading the segmentation performance of the model.

**Figure 1:**
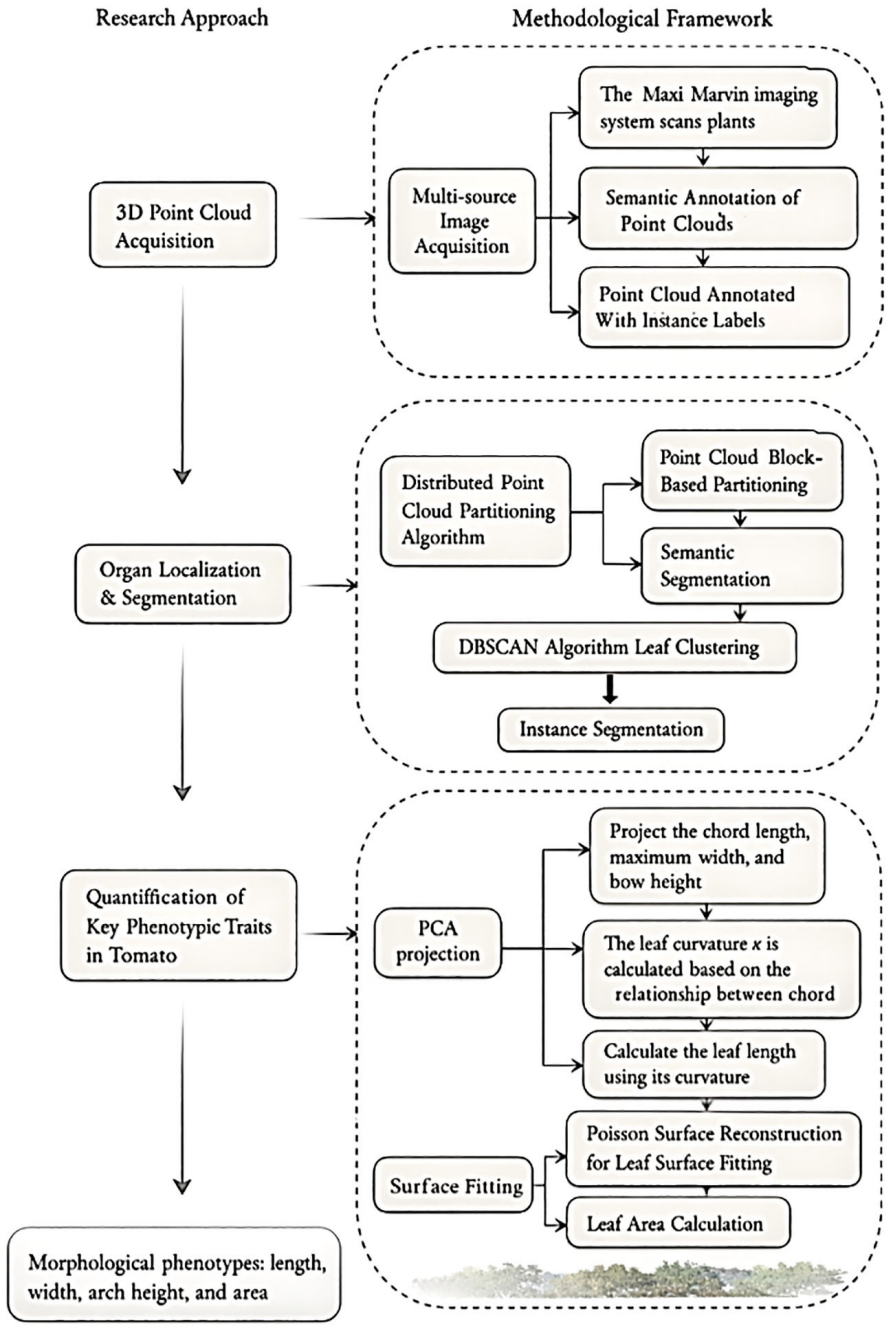
An overview of tomato leaf phenotyping with 3D distributed segmentation.

This study focuses on the quantification of key phenotypic traits of tomato leaves, including length, width, and area. To overcome the spatial resolution loss caused by the traditional segmentation network, a phenotyping pipeline based on distributed deep neural network is proposed, as illustrated in **Figure 1**. Different from the distributed segmentation method proposed by Xin et al., 2024, the proposed work divided the original point cloud into multiple non-overlapping subsets with an equal number of points in spatial order. This subset division strategy effectively avoids the uneven local point density caused by the non-replacement down sampling. As one of the effective and widely used point cloud segmentation networks (Guo et al., 2020), PointNet++ is adopted as the backbone of this study to perform parallel semantic segmentation on multiple subsets. The resulting outputs are then fused to produce the semantic representation of the entire point cloud. Subsequently, leaf instance segmentation is conducted using the density-based spatial clustering of applications with noise (DBSCAN), enabling the quantitative extraction of target phenotypic traits. The contributions of this research are listed as follows:

1. A distributed point cloud segmentation strategy was proposed to address the resolution loss caused by excessive down sampling. A systematic analysis of the potential factors influencing distributed segmentation performance was conducted.
2. A leaf trait quantification pipeline was proposed based on DBSCAN and 3D surface reconstruction algorithms, enabling a fully automated and high throughput quantification of key phenotypic parameters of leaves, including length, width, and area.

## 2 Materials and methods

This section is organized as follows: Section 2.1 introduces the imaging setup, dataset composition, and data annotation procedure; Section 2.2 describes the distributed point cloud segmentation strategy and model training process; Section 2.3 presents the leaf instance segmentation based on DBSCAN, followed by the quantification of leaf phenotypic traits in Section 2.4; Section 2.5 outlines the criteria used to evaluate the performance of the proposed work.

### 2.1 Data acquisition

**Figure 2:**
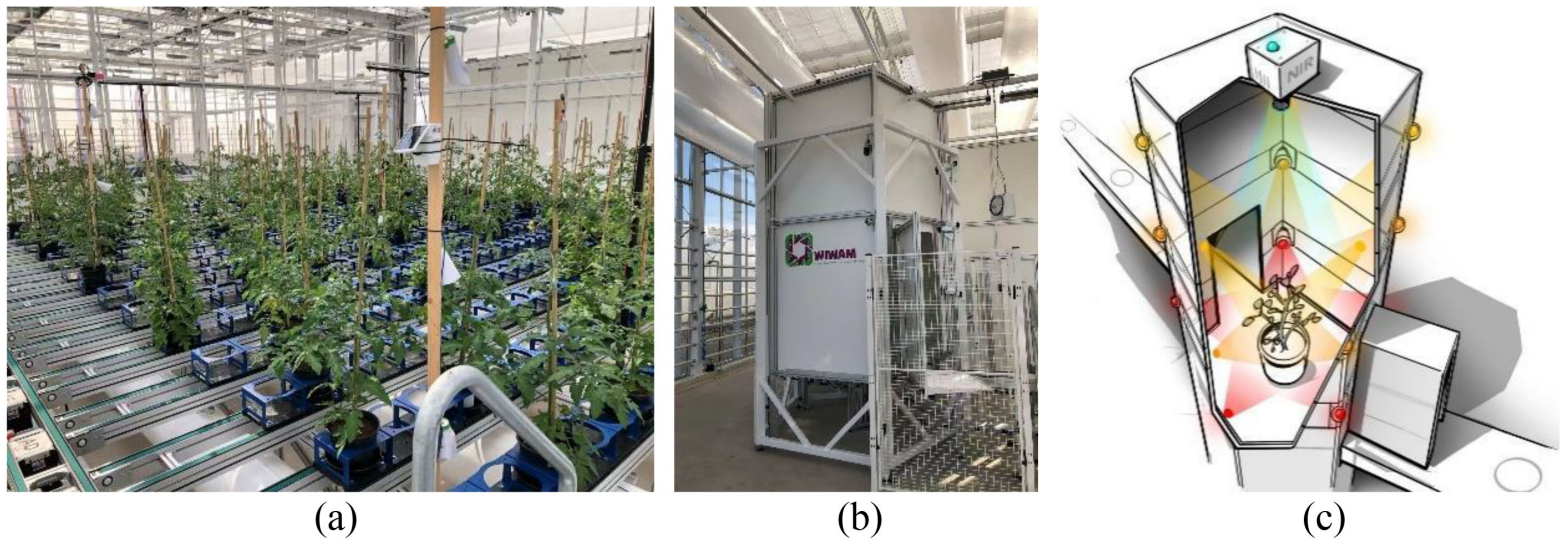
Data acquisition facilities, where **(a)** reveals the plant growing chamber of NPEC Greenhouse, **(b)** reveals the outline of Maxi-Marvin imaging system, and **(c)** is a legend of camera setup inside the imaging black box.

The dataset used in this paper is identical to the one presented in Xin et al. (2024). To facilitate understanding, an overview of the dataset is provided below. Three widely cultivated tomato (Solanum lycopersicum L.) varieties were selected, including 17 plants of Merlice, 20 plants of Brioso, and 8 plants of Gardener Delight. Plants were grown in a greenhouse of the Netherlands Plant Echo-phenotyping Centre (NPEC) [^1^], as shown in **Figure 2A**. Scanning was conducted during the second and third weeks after sowing, yielding 45 scans of tomato plants in total. During the second week, scanning was completed on 19 plants, comprising 7 plants of Merlice, 8 plants of Brioso, and 4 plants of Gardener Delight. In the third week, scanning was performed on the remaining 26 plants, including 10 plants of Merlice, 12 plants of Brioso, and 4 plants of Gardener Delight. These scans were partitioned into training set, validation set, and test set, containing 30, 10, and 5 scans respectively. To minimize varietal bias, the distribution of varieties within each sub-dataset was maintained in proportion to that of the overall dataset.

**Figure 3:**
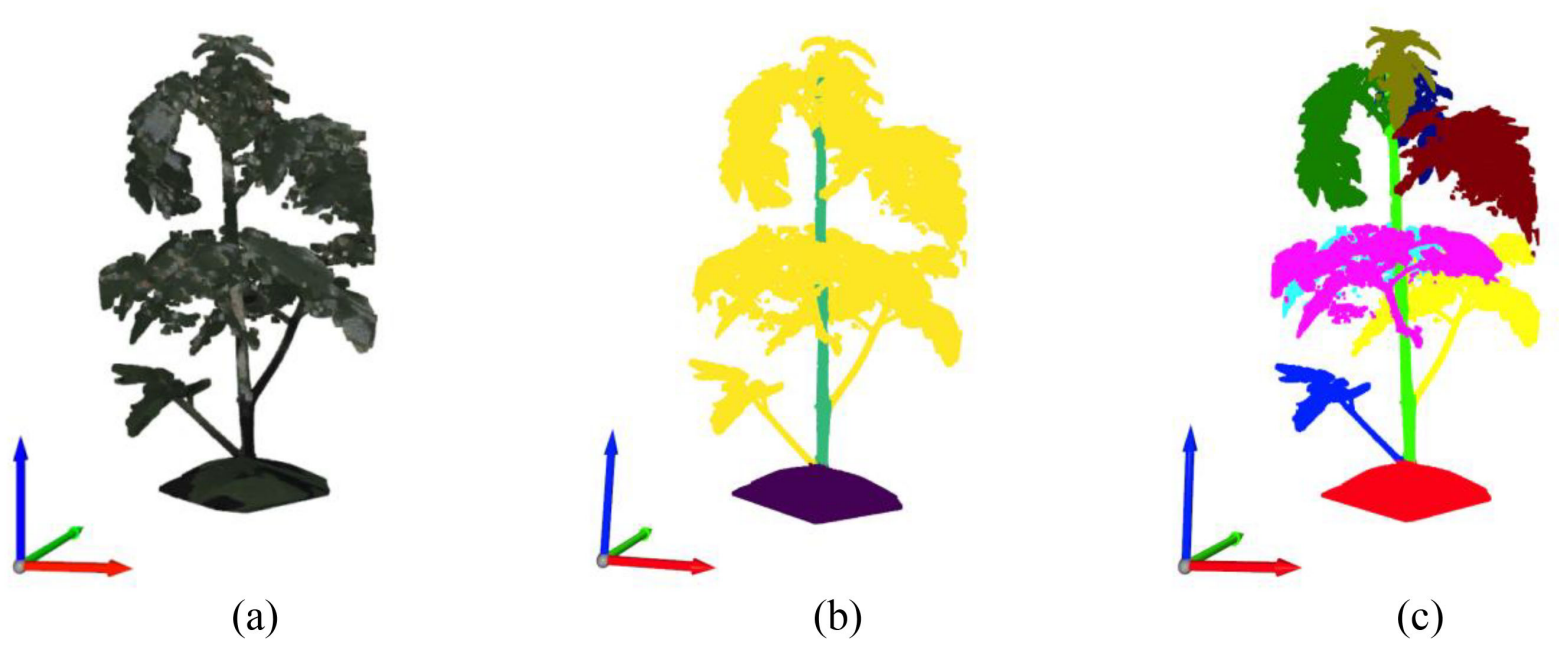
An example of raw point cloud and manual annotation. **(a)** shows an example of colored point cloud, where the visualized color is derived from the median values of the R, G, and B channels captured by 15 cameras; **(b)** displays the corresponding semantic labels, where the soil base, main stem, and leaves are marked by purple, green, and yellow respectively; **(c)** presents the leaf instance labels, where individual leaf instances are marked by different colors.

Scanning was performed with an imaging setup in NPEC greenhouse, naming Maxi-Marvin, as shown in **Figures 2B, C**. This device employs a pentaprism-shape imaging black box, with the target plant placed at the center of the black box. Three RGB cameras with a resolution of 1920×1080 were uniformly mounted on each of the five edges, resulting in 15 cameras in total. During each snapshot, 15 images of the target plant were captured simultaneously from different perspectives. A 3D voxel space with a dimension of 40cm×40cm×70cm and a spatial resolution of 1mm^3^ was constructed at the center of the imaging black box. The point cloud of the target plant was reconstructed using a shape-from-silhouette method proposed by Golbach et al. (2016). The final point cloud contains 48 channels, including three channels of spatial information, and 45 channels of RGB color information captured by 15 cameras respectively. **Figure 3A** shows an example of RGB point cloud acquired by the Maxi-Marvin system.

The point cloud annotation was conducted in two aspects: semantic labeling and instance labeling. The semantic annotation categorized points into three classes: soil, main stem, and leaves, as shown in **Figure 3B**. Based on semantic annotation, instance annotation further divided the leaf points into individual instances and assigned instance labels in spatial bottom to top order (**Figure 3C**) [^2^].

Destructive measurements of leaf length, width, and area were conducted right after the imaging process. Here, leaf length and width were measured manually using a ruler with 0.5mm graduations. Leaf area was measured using LI-3000C leaf area meter after leaves were detached from the plant.

### 2.2 Distributed semantic segmentation strategy

The proposed work employed a deep-learning-based segmentation to localize organs within a point cloud according to their types. Deep neural networks typically require inputs with consistent dimensions. Traditional down sampling satisfies this requirement at the cost of reduced resolution and loss of target detail, which introduce errors to the following phenotyping process. Alternatively, distributed neural networks partition the point cloud into smaller subsets with a consistent point number, and processes these subsets in parallel. Final result is presented by fusing the outputs of individual subsets together. This strategy minimizes information loss while satisfying the input dimension requirements of neural network.

#### 2.2.1 Subset division

**Figure 4:**
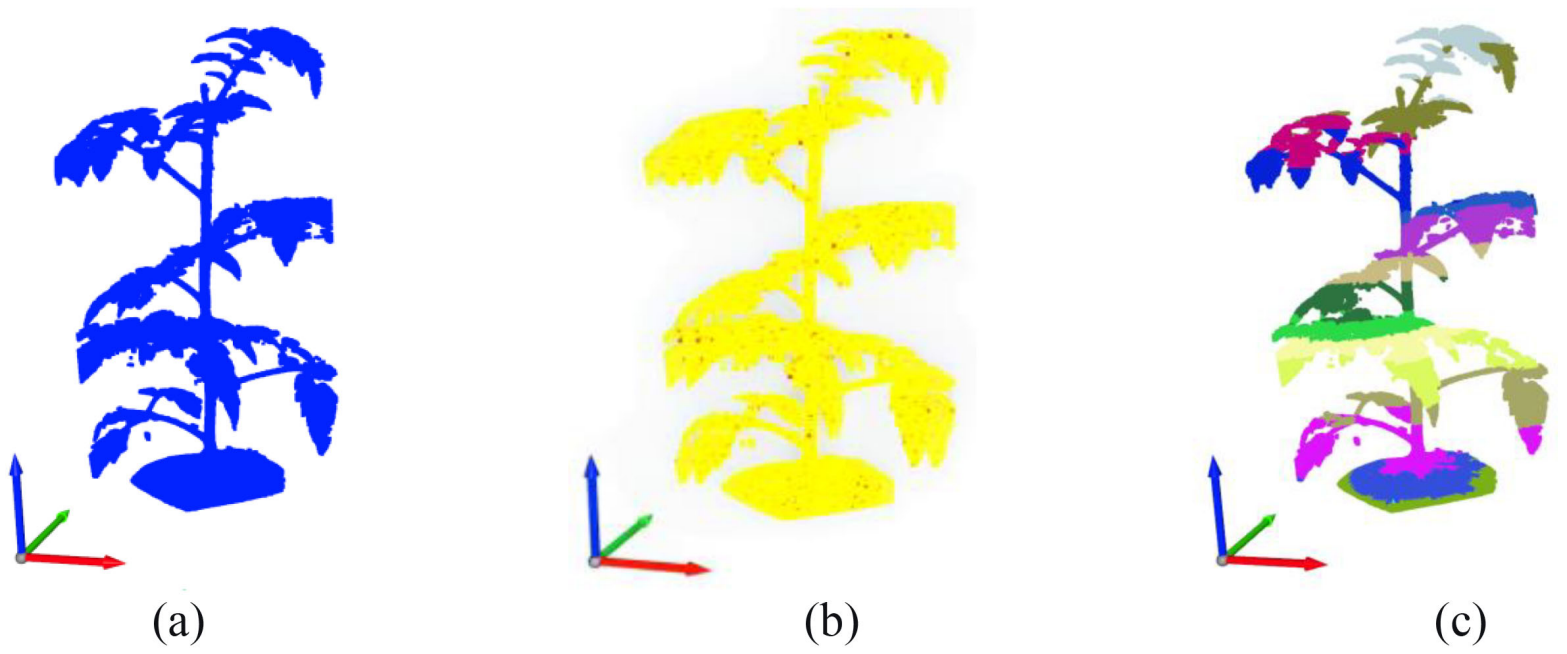
Legend of subset division. **(a)** shows the original point cloud containing 26,903–5 points; **(b)** reveals the excessive point removal with a block size of 20,000, where the points being removed are marked by red; **(c)** shows the final subset division result.

**Figure 5:**
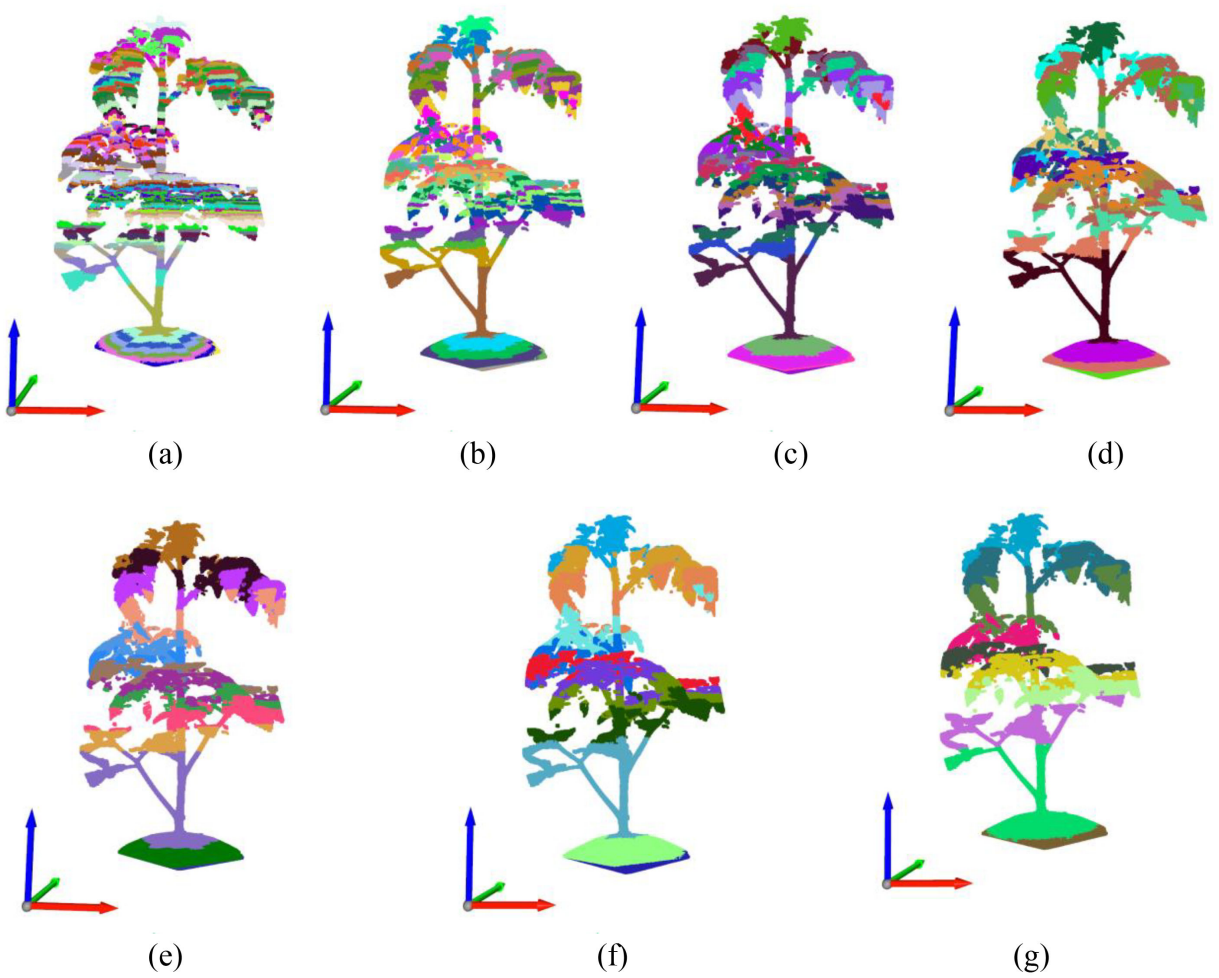
Subset division results with block sizes of **(a)** 5,000, **(b)** 10,000, **(c)** 15,000, **(d)** 20,000, **(e)** 25,000, **(f)** 30,000 and **(g)** 35,000 respectively.

Before training, the original point cloud was divided into multiple subsets in spatial top-down order without overlapping. Since the numbers of points vary among point clouds, we randomly excluded excessive points to ensure each point cloud could be evenly divided into subsets with the pre-defined block size. For example, **Figure 4a** reveals a tomato plant point cloud containing 461,725 points. By employing a block size of 20,000, 461,725% 20,000 = 1725 points are randomly selected and removed from the original point cloud, as shown in **Figure 4b**. Here, “%” is a modulo operator. The final subset partition result is shown in **Figure 4c**, where individual subsets were marked by a unique color. To improve the clarity, the proposed subset division strategy is summarized in **Algorithm 1**. **Figure 5** further illustrates the subset partition results with block size of 5,000, 10,000, 15,000, 20,000, 25,000, 30,000 and 35,000 respectively.

Algorithm 1

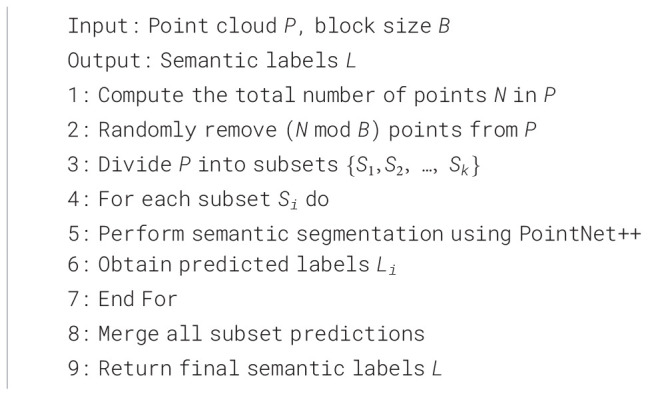

#### 2.2.2 Network backbone and model training

By taking each point cloud subset as input, organ semantic segmentation was performed using PointNet++ model. PointNet++ builds a local feature extractor that combines shared Multiple Layer Perceptions (MLPs) with pooling layers to aggregate permutation-invariant features. To capture hierarchical features, set abstraction layers that perform sampling and grouping on top of the local feature extractor are further introduced. The resulting higher-level features are allocated back to individual points via feature propagation based on spatial interpolation. The propagated feature vectors are fed into a sequence of fully connected layers, yielding a 1×3 output vector for each point representing the probabilities corresponding to respective classes. The predicted annotation is obtained by selecting the class with the maximal probability. During the training process, batch size was set to five to prevent memory runout, and the number of training epoch was selected as 150 to reduce the overfitting risk. The segmentation result for the complete point cloud was finally presented by merging the segmentation outputs of individual subsets according to their spatial order.

### 2.3 Leaf instance segmentation

To facilitate the following phenotypic trait calculations, a leaf instance segmentation was further performed based on the semantic segmentation result from Section 2.2. To avoid the overfitting problem of instance segmentation network caused by insufficient training data, this study proposed a deep-learning-free segmentation pipeline for leaf instances. Points of the same leaf instance cluster densely and continuously, whereas different instances are separated by low-density regions and clear spatial gaps. Inspired by Xin et al. (2024), a Density-Based Spatial Clustering of Applications with Noise (DBSCAN) algorithm was employed to group leaf points into instances.

DBSCAN clusters a point cloud by identifying core points, border points, and noise points. A point is recognized as a core point if it satisfies a density criterion: the number of points within its *ϵ*-neighborhood is larger than a predefined threshold $N_{\min}^{C}$, where *ϵ* denotes the radius of the query ball. Points that do not meet this criterion but lie within the neighborhood of an existing core point are recognized as border points. Noise points are neither core points nor density-reachable from any core point. Cluster expansion begins with an identified core points and iteratively aggregates all density-reachable core and border points. The final clustering result is obtained by collecting the points assigned to each expanded cluster. To suppress small and fragmented clusters corresponding to invalid leaf instances, a noise filter is further applied to the initial clustering results. Specifically, clusters whose point counts fall below a predefined outlier threshold $N_{\min}^{N}$ are treated as spurious low-density clusters and are subsequently discarded. Hyper parameters of *ϵ*, $N_{\min}^{C}$ and $N_{\min}^{N}$ were optimized by evaluating the instance segmentation performance over the validation set. Corresponding values were finally selected as *ϵ* = 10 mm, $N_{\min}^{C}=50$, and $N_{\min}^{N}=5,000$ respectively. The proposed leaf instance segmentation process is summarized in **Algorithm 2** for clarity.

Algorithm 2

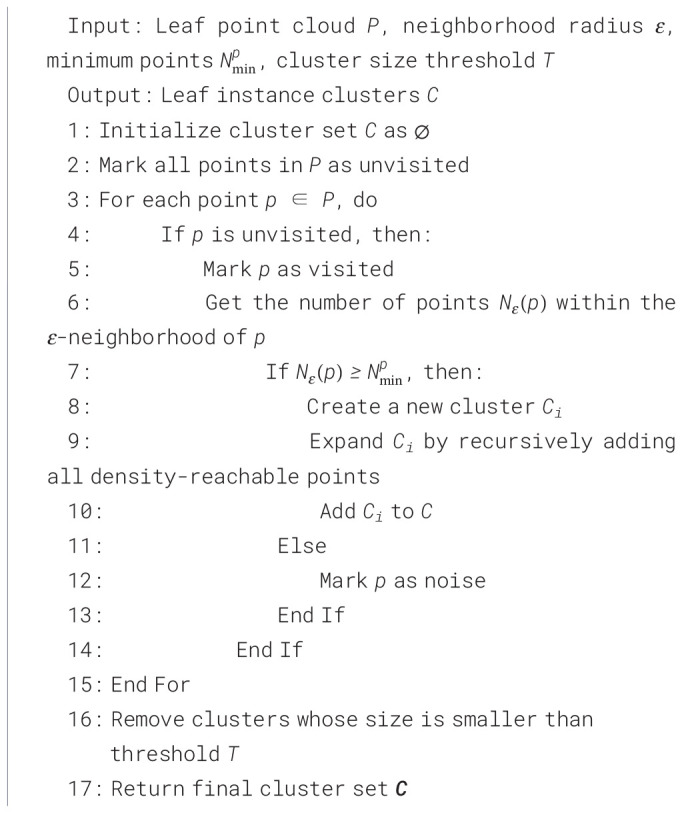

### 2.4 Quantification of leaf phenotypic traits

**Figure 6:**
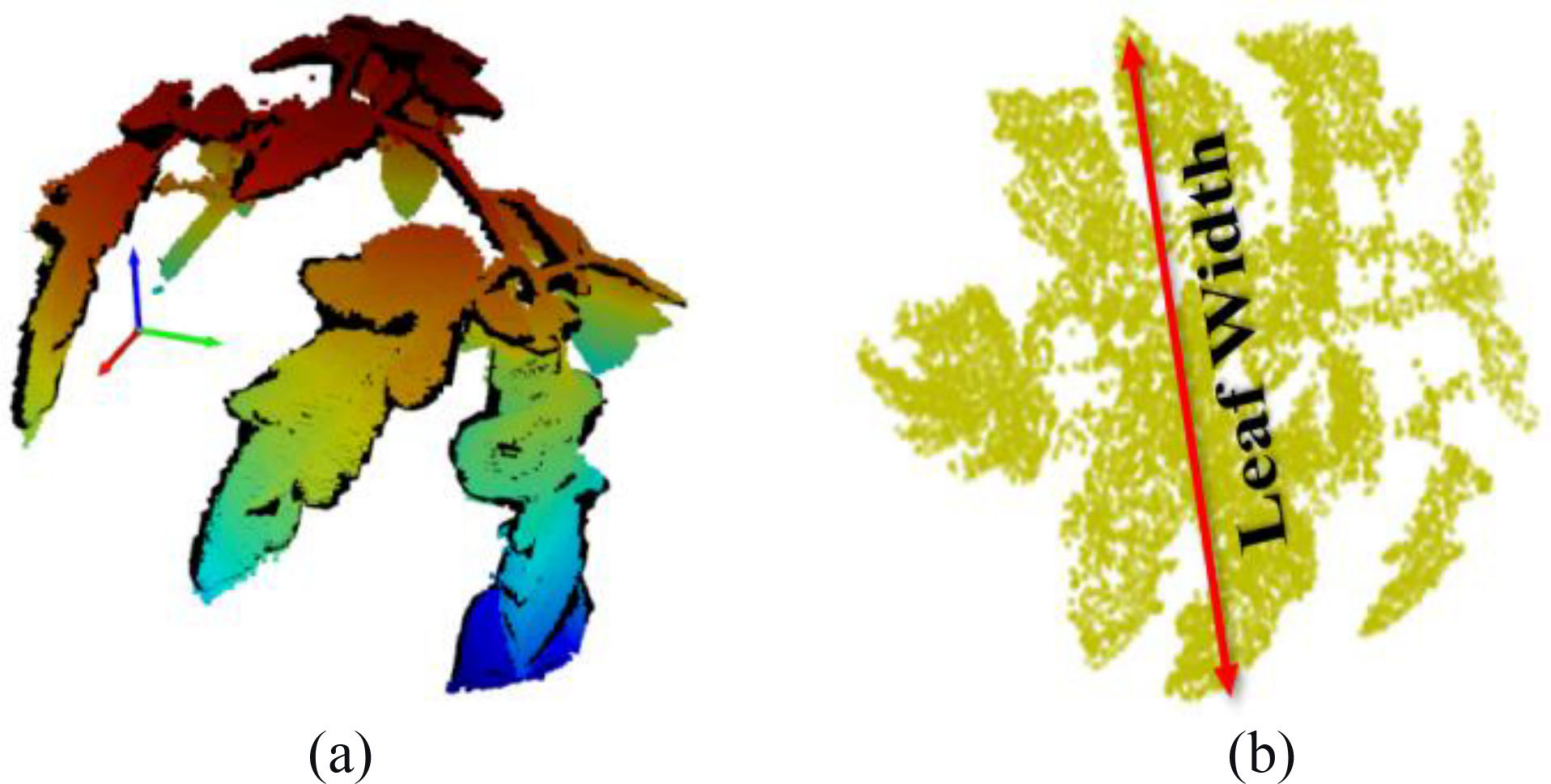
Leaf width quantification based on principal component analysis (PCA).

**Figure 7:**
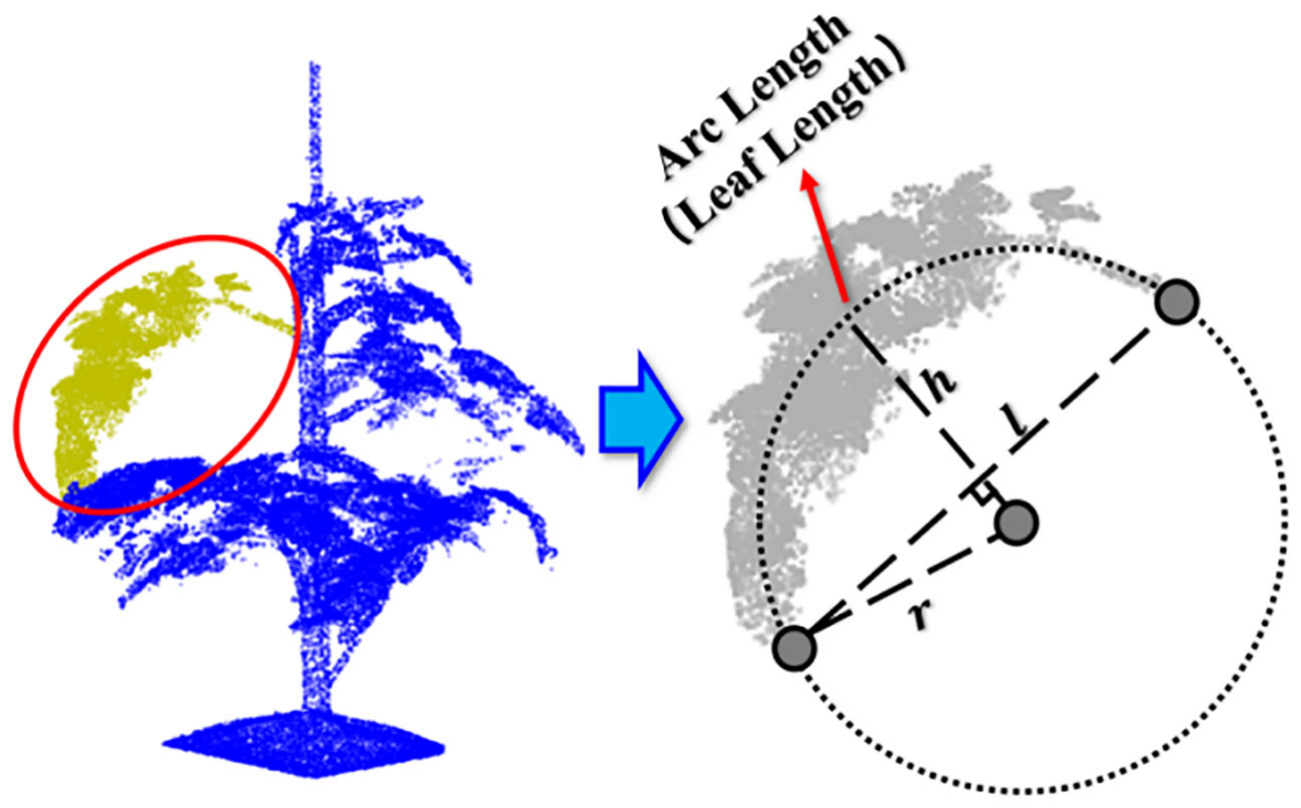
Leaf length quantification in the case of large curvature based on circle fitting.

The leaf width is defined as the maximum distance perpendicular to the main rachis, as shown in **Figure 6**. To quantify leaf width, Principal Component Analysis (PCA) was applied to the leaf points, followed by fitting a 3D oriented bounding box [^3^]. Leaf width was then determined as the side length of the oriented bounding box aligned with the second principal axis. Leaf length is defined as the distance along the leaf surface between the petiole base and the leaf apex. However, leaves of tomato plants commonly indicate arched shapes, with surfaces exhibiting obvious curvature, as shown in **Figure 7**. In this case, taking the straight-line distance between the petiole base and the leaf apex as the leaf length may introduce significant errors, and this error increases as the increment of leaf curvature. Therefore, a geometric approximation was proposed to quantify leaf length with significant curvature. The leaf shape can be approximated by a circle with radius *r*, as shown in **Figure 7**. Here, chord length *l_c_* refers to the Euclidean distance between the petiole base to the leaf tip, and bow height *h* refers to the distance from the highest point of the leaf to the principal axis. In this manner, the length of the leaf can be approximated using the arc length *l_a_*:

(1) $$l_{a}=r\cdot 2\theta$$

where *θ* refers to the half-central angle subtended by arc *l_a_*:

(2) $$\theta =\sin^{-1}\frac{l_{c}}{2r}$$

The arc length *l_a_* can be calculated by substituting Equation 2 to Equation 1, yielding Equation 3:

(3) $$l_{a}=2r\sin^{-1}\frac{l_{c}}{2r}$$

The radius *r* of the fitted circle can be derived from Equations 4, 5:

(4) $$r^{2}={\left(r-h\right)}^{2}+{\left(\frac{l_{c}}{2}\right)}^{2}$$

(5) $$r=\frac{l_{c}^{\;2}}{8h}+\frac{h}{2}$$

To balance computational efficiency and measurement accuracy, we introduced a trade-off strategy based on leaf curvature *κ*, whose calculation is given in Equation 6:

(6) $$\kappa =\frac{1}{r}$$

When *κ* < 1/3, the leaf length was taken as the chord length *l_c_* directly [^4^]; Otherwise, the arc length *l_a_* calculated from Equation 3 was used to approximate the leaf length instead.

**Figure 8:**
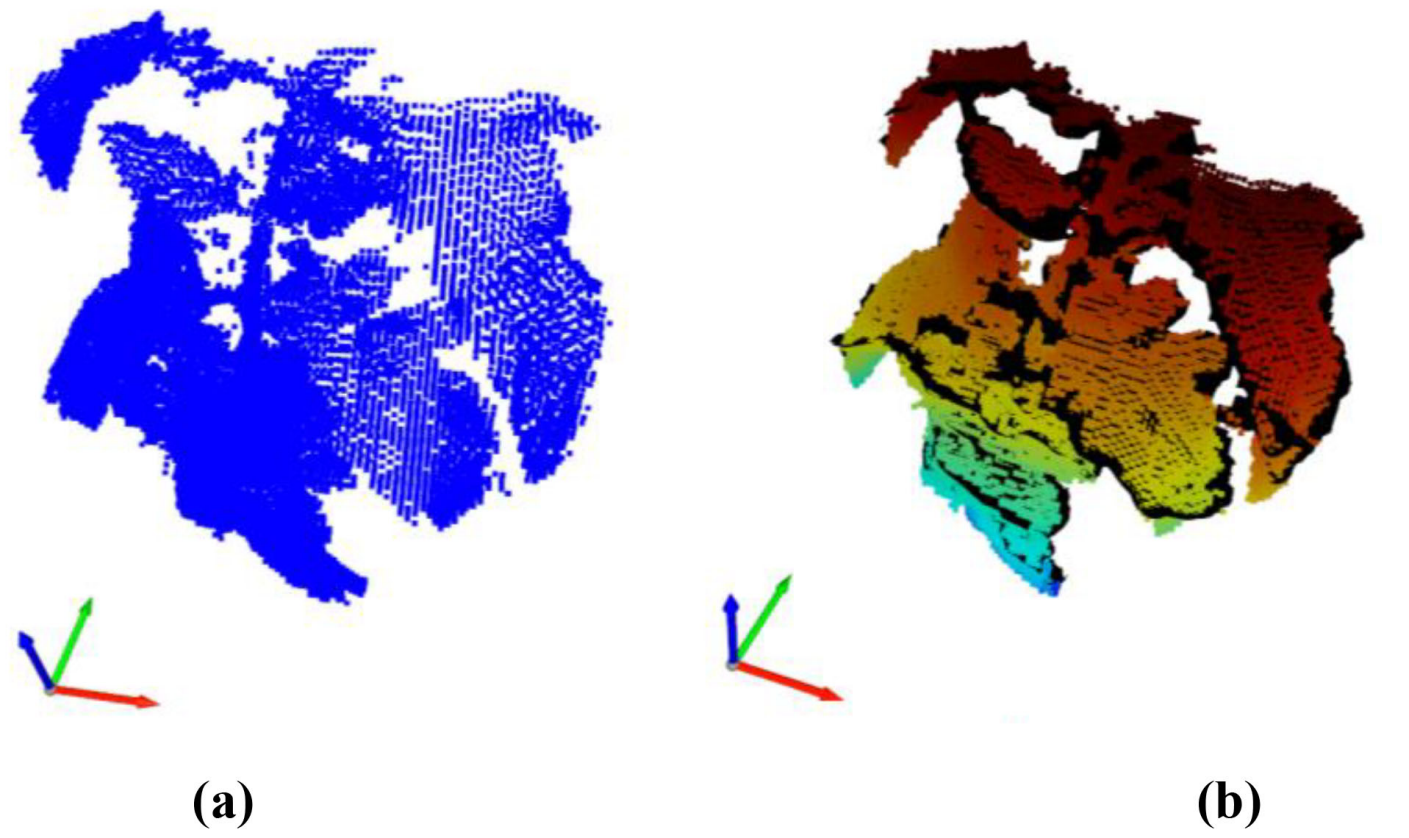
Leaf surface fitting result: **(a)** segmented leaf point cloud and **(b)** triangular surface mesh with Poisson reconstruction.

To acquire leaf area, Poisson reconstruction was employed to fit a smooth surface for each leaf instance and represent it mathematically. Hyper-parameters including reconstruction depth and extension ratio were selected based on the reconstruction performance over the validation set. Here, the reconstruction depth determines the sampling resolution of the leaf surface within the Poisson function, directly affecting the accuracy of morphological features such as leaf margin contours and curvature in the vein regions, which was set to eight to retain target details and minimize noise. An extension ratio of 1.1 was applied to the surface boundary to mitigate edge shrinkage and preserve the complete contours of the edges. The reconstructed leaf surface was represented by multiple triangular patches, which was revealed in **Figures 8a** and **b**. Leaf area was then calculated by summing the areas of all constituent triangular patches. The procedure for leaf phenotypic trait quantification is summarized in **Algorithm 3**.

Algorithm 3 **Leaf phenotypic trait quantification.**

Input: Leaf point cloud *P*Output: Leaf length *L*, width *W*, and area *A*1: Perform Principal Component Analysis (PCA) on *P*2: Determine the principal axis direction3: Project points onto the principal axis4: Compute chord length *c* between leaf base and apex5: Compute bow height *h* from projection range6: Estimate curvature *k* based on *c* and *h*7: If *k* is smaller than a predefined threshold, then8: Set leaf length *L* as *c*9: Else10: Compute arc length *L* based on curvature11: End If12: Compute leaf width *W* using oriented bounding box13: Perform Poisson surface reconstruction on *P*14: Represent the surface using triangular meshes15: Compute leaf area A by summing triangle areas16: Return L, W, and A

### 2.5 Evaluation metrics

#### 2.5.1 Semantic and instance segmentation

The experiments were implemented on a desktop computer with an Intel Core i5-12400F CPU, 32 GB RAM, and an NVIDIA GeForce RTX 3050 GPU (6 GB VRAM). To further evaluate the transferability of the proposed distributed segmentation strategy, PointTransformer and PointConv were also employed as backbone models for parallel comparison. The performance of organ semantic and instance segmentation was evaluated through Intersection over Union (IoU) and *F*1 score respectively. With respect to a semantic or instance class *i*, IoU measures the proportion of points whose predicted labels exactly match with the manual annotation, calculated from Equation 7:

(7) $$IoU=\frac{TP}{TP+FP+FN}$$

Here, *TP* refers to true positives, denoting the number of points being predicted correctly with respect to class *i*; *FP* represents false positives, denoting the number of points being assigned to class *i* while they are actually not; *FN* refers to false negatives, indicating the number of points whose ground truth annotation fall into class *i*, but were assigned to other classes.

Given the above definition, *F*1 score is calculated from Equation 8:

(8) $$F1=2\times \frac{Precision\times Recall}{Precision+Recall}$$

where precision and recall are obtained from Equations 9, 10 respectively:

(9) $$Precision=\frac{TP}{TP+FP}$$

(10) $$Recall=\frac{TP}{TP+FN}$$

#### 2.5.2 Quantification of leaf traits

This study introduces Mean Absolute Error (MAE) and Root Mean Squared Error (RMSE) as evaluation metrics for leaf trait quantification, calculated from Equations 11, 12 respectively:

(11) $$AE=\frac{1}{n}\sum_{i=1}^{n}\left|y_{i}-\hat{y_{i}}\right|$$

(12) $$RMSE=\sqrt{\frac{1}{n}\sum_{i=1}^{n}{\left(y_{i}-\hat{y_{i}}\right)}^{2}}$$

Here, *n* represents the total number of samples, *y_i_* denotes the ground truth value of the *i^th^* sample, while $\hat{y_{i}}$ refers to the corresponding predicted value.

## 3 Results and discussion

### 3.1 Semantic segmentation

#### 3.1.1 Effect of block size on semantic segmentation performance

**Figure 9:**
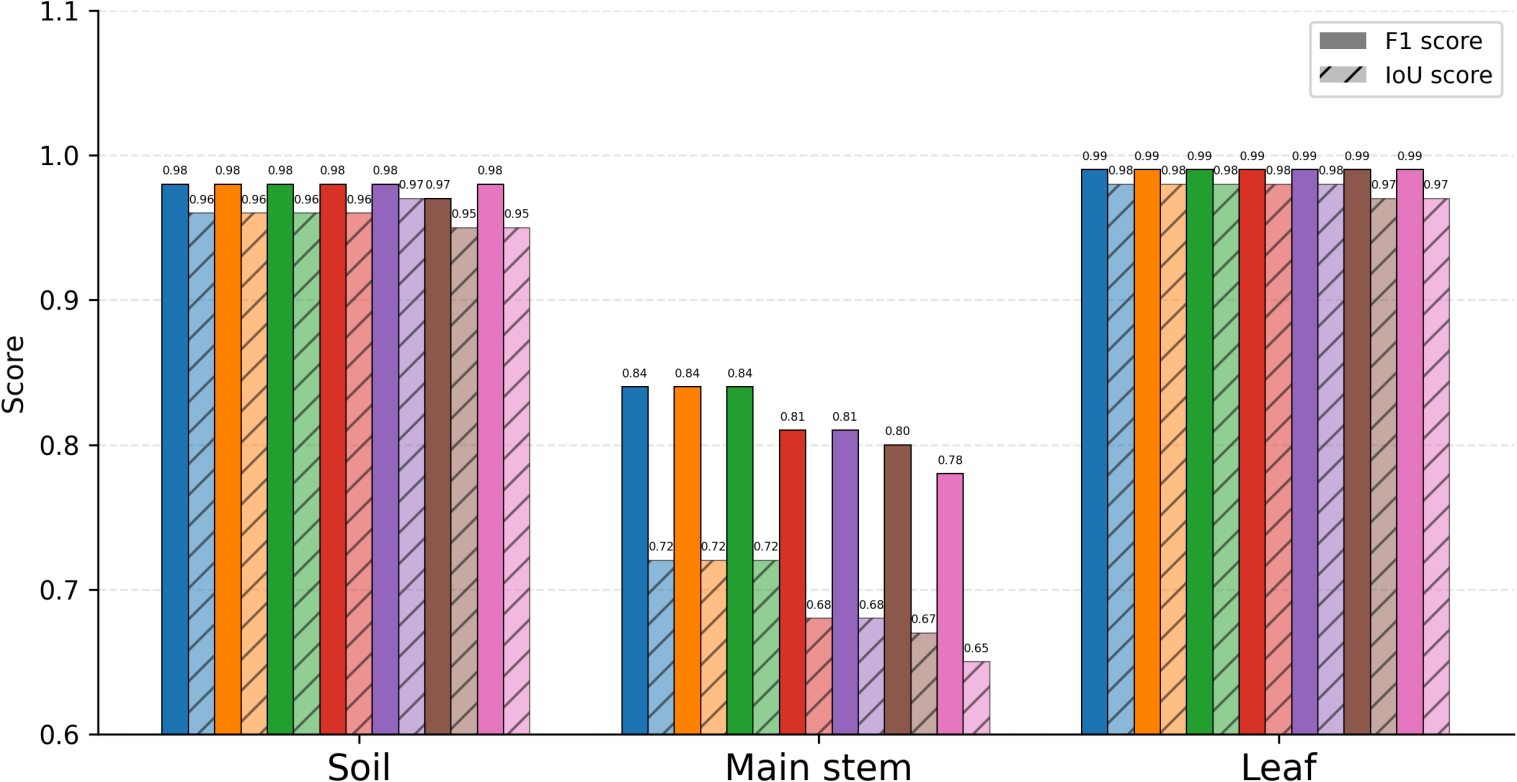
Performance comparison of semantic segmentation under different point cloud block sizes. The Intersection over Union (IoU) and *F*1 scores are reported for three semantic classes: soil, main stem, and leaf. Results are evaluated using seven block sizes, namely 35,000, 30,000, 25,000, 20,000, 15,000, 10,000, and 5,000 points. Solid bars denote the *F*1 score, while hatched bars indicate the IoU score. Color coding corresponds to block size: blue represents a block size of 35,000 points, orange represents 30,000 points, green represents 25,000 points, red represents a block size of 20,000 points, purple represents 15,000 points, brown represents 10,000 points, and pink represents 5,000 points.

The IoU and *F*1 scores for different classes under various block sizes are shown in **Figure 9**. The evaluation results indicate that block size did not significantly affect semantic segmentation performance. Nevertheless, exceptionally small block sizes may disrupt the integrity of object features, thereby hindering feature learning and resulting in a slight decline in segmentation performance.

Based on the *F*1 scores and IoU metrics, the stem class exhibited substantially lower segmentation performance than the soil and leaf classes. This was mainly due to the loss function used - average cross entropy - was sensitive towards class imbalance. In the dataset used in this study, leaf points account for the largest proportion (82.27%), followed by the main stem class (15.48%), while the stem class accounts for only 3.15%, which is significantly lower than the other categories. Under such an imbalanced distribution, to minimize the loss function, the neural network placed greater emphasis on the more frequent classes during training, while underemphasizing the stem class. To address this issue, future work will incorporate focal loss (Lin et al., 2017), which reduces the contribution of easily classified samples to the overall loss while increasing the weight assigned to underrepresented classes. Another potential reason giving rise to this problem was the insufficient training data. Since the annotation process of 3D point clouds is laborious and time consuming, a training set with enough samples is usually not available. The dataset used in this paper contains only 45 samples, and this limited diversity increases the risk of overfitting. Local augmentation of plant organs has been shown to effectively mitigate network overfitting (Xin et al., 2023), and can be applied in the future to enhance dataset diversity.

A parallel comparison was performed with the distributed segmentation strategy proposed by Xin et al. (2024). Their approach reported *F*1 scores of 0.98, 0.65, and 0.96 for the soil, stemwork, and leaf classes respectively. The proposed distributed segmentation method achieved the best performance at a block size of 30,000, with corresponding *F*1 scores of 0.98, 0.74, and 0.99 respectively. The most pronounced improvement was observed in stemwork segmentation, where the *F*1 score increased from 0.65 to 0.74. Since Xin et al. (2024) employed non-replacement resampling for subset division, the local density of points varied across subsets, rendering density an unreliable feature for segmentation. In contrast, the proposed spatial blockization approach preserved a relatively stable local point density, which may account for its better performance. As described in Section 2.1, the dataset used in this study is identical to that of Xin et al. (2024), making the comparison valid and fair.

Although the proposed method indicated a slightly better performance, it also introduced additional computational overhead. The non-overlapping re-sampling strategy proposed by Xin et al. (2024) takes 38.69 minutes for model training, while the proposed distributed segmentation strategy consumes 80.33 minutes. However, the improved segmentation performance, particularly in stemwork regions, substantially enhances following phenotyping performance, which outweighs the extra computational cost during training process. In aspect of inference, their method takes 18.3 seconds to process a single point cloud, while the proposed approach requires 16.0 seconds - the two results are nearly comparable. Here, it is worthwhile to mention that the computational platform used in Xin et al. (2024) was an Intel Xeon E-2276M (2.8GHz) CPU and a NVIDIA Quadro RTX 5000 Max-Q (16GB) GPU, which was different from the one used in the proposed work (Section 2.5.1). This may give rise to certain deviations when comparing the training and inference time.

#### 3.1.2 Comparison with alternative network architectures

Two widely adopted 3D segmentation models - PointTransformer and PointConv - were incorporated as backbones for additional validation experiments to further assess the transferability of the proposed distributed segmentation strategy. The results indicated that PointTransformer achieves an overall IoU of 0.84 and an *F*1 score of 0.91, with a training time of 55.47 minutes. PointConv achieves an IoU of 0.81 and an *F*1 score of 0.89, with a training time of 51.65 minutes. Comparing with the above backbone models, PointNet++ achieves an IoU of 0.96 and an *F*1 score of 0.98 with a training time of 80.33 minutes. Although PointNet++ requires a relatively longer training duration, this computational overhead is fully offset by its improvements in segmentation accuracy and robustness.

### 3.2 Instance segmentation

**Figure 10:**
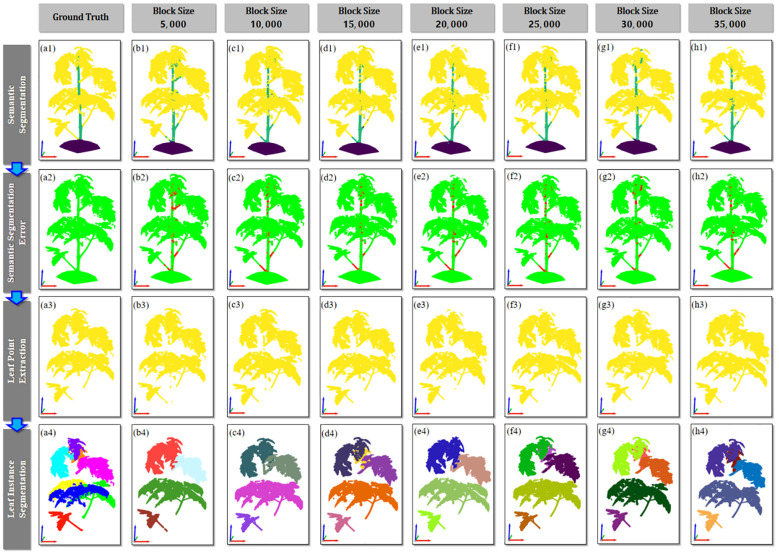
Results of tomato plant organ semantic segmentation and leaf instance segmentation under seven different block sizes compared with the ground-truth data. The first column shows the manually annotated ground-truth data, whereas columns two - eight present the segmentation results obtained using block sizes ranging from 5,000 to 35,000 points. The first row illustrates the semantic segmentation results, where different colors represent different plant organs. The second row shows the semantic segmentation errors, in which red points indicate misclassified points relative to the ground truth. The third row presents the leaf point clouds extracted from the semantic segmentation results. The fourth row displays the final leaf instance segmentation results, where different colors distinguish individual leaves.

Block size is a key hyperparameter in semantic segmentation and intermediately influences instance segmentation performance. We quantitatively evaluated instance segmentation performance under different block-size settings using metrics of *F*1 score, IoU, and mis-segmentation rate. Here, mis-segmentation rate refers to the ratio of unassigned leaf number relative to the total number of ground-truth leaf number. **Figure 10** visualizes the instance segmentation performance with respect to various block sizes, and **Table 1** presents the corresponding quantitative evaluation results.

**Table 1:** Evaluation results for instance segmentation.

Evaluation metrics	Block size of 35,000	Block size of 30,000	Block size of 25,000	Block size of 20,000	Block size of 15,000	Block size of 10,000	Block size of 5,000
IoU	0.70	0.74	0.68	0.65	0.60	0.66	0.64
*F*1 Score	0.73	0.77	0.74	0.67	0.63	0.68	0.66
mis-segmentation rate	17.39%	14.49%	17.39%	17.39%	21.74%	14.49%	17.39%

As shown in **Table 1**, the IoU and *F*1 scores of all groups are generally above 0.60, while the mis-segmentation rates range between 14.39% and 17.39%, except for the block size of 15,000. The model achieves the best performance at a block size of 30,000, with an *F*1 score, IoU, and mis-segmentation rate of 0.74, 0.77, and 14.49% respectively. This indicates that a block size of 30,000 effectively preserves the key topological structures and morphological features required for semantic segmentation, while avoiding the excessive computational cost associated with overly large blocks. Although the block sizes of 25,000 and 35,000 points exhibit slightly higher mis-segmentation rates than the block size of 30,000, their IoU and *F*1 scores remain at relatively high levels. The block size of 10,000 also demonstrates competitive performance, with an *F*1 score and IoU of 0.68 and 0.66 respectively, and a mis-segmentation rate identical to that of the optimal block size of 30,000 (14.49%). In contrast, the instance segmentation performance decreases for the block size of 20,000 and block size of 5,000, both yielding a mis-segmentation rate of 17.39%. Notably, the block size of 15,000 yields the worst segmentation performance, with a mis-segmentation rate as high as 21.74%. This is mainly attributed to the difficulty in extracting discriminative features during the semantic segmentation stage under this block size.

**Figure 11:**
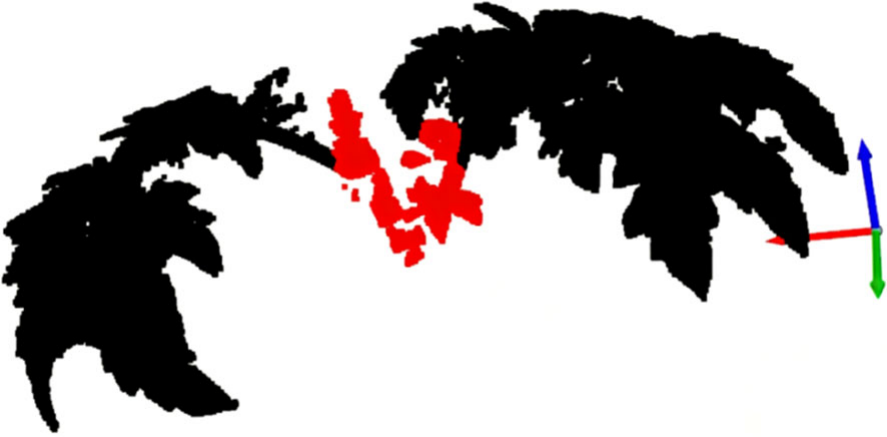
False connection between adjacent leaf instances.

Although block size affects instance segmentation results to some extent, the overall instance segmentation performance is still relatively low. This was primarily due to the absence of density gaps that DBSCAN relies on for clustering. For those long and drooping leaves, substantial overlapping and crossover cause multiple leaf instances to come into contact, with no clear density gaps in between adjacent leaves in the point cloud. In this case, DBSCAN may classify multiple distinct leaves as a single instance, leading to significant under-segmentation problems. On the other hand, errors introduced in the preceding semantic segmentation stage also affect the instance segmentation performance as well. During semantic segmentation, certain points belonging to the main stem were incorrectly assigned to the leaf class. In the subsequent instance segmentation stage, these misclassified stem points create “false connections” between adjacent leaves, as shown in **Figure 11**. As a result, the lack of a clear density gap caused DBSCAN to merge multiple leaves into a single instance, directly reducing instance segmentation accuracy.

A control experiment was conducted to further evaluate the impact of semantic errors on instance segmentation performance. Based on manually annotated semantic labels, stem and soil points were excluded from the point cloud, leaving only leaf points. DBSCAN was then applied to the resulting leaf points, and the clustering result was compared with manually annotated leaf instances. The control experiment achieved an IoU of 0.73 and an *F*1 score of 0.76. While “false connections” between adjacent leaves were avoided in this control experiment, DBSCAN still struggled to achieve accurate clustering in structurally complex regions where leaves interlace and overlap. These results suggest that eliminating semantic segmentation errors alone is insufficient to resolve the challenges in leaf instance segmentation.

### 3.3 Quantification of leaf phenotypic parameters

#### 3.3.1 Leaf length and width

To ensure that leaf phenotypic traits were quantified correctly, under-segmented leaf instances were excluded, and only structurally complete leaf instances were retained. The average errors of predicted leaf length and width are revealed in **Table 2**.

**Table 2:** Quantification results of leaf length and leaf width.

Leaf	Unit (cm) \ Indicator	Block size of 35,000	Block size of 30,000	Block size of 25,000	Block size of 20,000	Block size of 15,000	Block size of 10,000	Block size of 5,000
Length	MAE	2.16	2.09	2.35	2.28	2.10	2.32	2.80
	RMSE	6.05	5.85	6.64	6.03	5.94	6.57	7.93
	Accuracy	91.71%	91.98%	90.98%	91.25%	91.94%	91.10%	89.26%
Width	MAE	1.65	1.78	1.73	2.02	1.40	2.06	1.83
	RMSE	5.49	5.17	5.15	5.71	5.10	5.82	5.18
	Accuracy	93.20%	92.66%	92.87%	91.67%	94.19%	91.51%	92.45%

Block size of 30,000 yields the lowest Mean Absolute Error (MAE) and Root Mean Square Error (RMSE) of 2.09cm and 5.85cm respectively for leaf length quantification, with an accuracy of 91.98%. Similarly, block size of 15,000 achieves the lowest MAE (1.40cm) and RMSE (5.10cm) for leaf width quantification, with an accuracy of 94.19%. Since under-segmented leaf instances were strictly excluded prior to the phenotyping process, the remaining valid leaf samples demonstrated relatively lower errors in both length and width estimation.

The performance of leaf width estimation is generally poorer than that of leaf length. The reasons are two folds. Firstly, segmentation errors occurring around the junction between the leaf vein and the main stem led to overestimation or underestimation of leaf length. Quantitative evaluation indicates that the *F*1 scores and IoU for main stem segmentation are lower than those for leaves and soil classes, giving rise to two types of segmentation errors at the junction region: i) points belonging to the main stem are mis-classified as leaf vein; and ii) points belonging to the leaf vein are mis-classified as the main stem, which subsequently excluded during the leaf point extraction process. Since leaf length is computed by taking the petiole base and the leaf apex as the reference points, the above segmentation errors alter the measurement reference points for leaf length, reducing the accuracy of length measurements. However, leaf width calculations do not suffer from these segmentation errors, thus maintaining higher precision. On the other hand, the geometrical approximation method during leaves length quantifications may also introduce errors. In the proposed method, leaf shape was approximated as a circle, and leaf length was represented by the corresponding arc length. However, most leaves have irregular shapes and pronounced curvature, which deviate from ideal circular arcs. This may lead to deviations in leaf length estimation. In contrast, leaf width estimation was less affected by this issue because it was calculated as the side length of the oriented bounding box aligned with the second principal axis. Since leaves usually bend along the direction of the veins, curvature along the second principal axis can be largely ignored, making leaf width estimation less sensitive to shape irregularities. Future work will focus on introducing more suitable shape models for leaf length approximation, such as ellipse arcs.

#### 3.3.2 Leaf area

The evaluation results of leaf area estimation are presented in **Table 3**. The leaf area estimation accuracy at block size of 30,000 achieved the best performance among the seven groups. With this configuration, the model attained an estimation accuracy of 89.67% for leaf area. There was no significant difference in estimation accuracy among the block size of 35,000, 20,000, and 15,000, indicating that as long as the block size is sufficient to preserve spatial continuity, the estimation remains stable. Within this range of segmentation sizes, the algorithm maintained stable estimation performance. Although the mean average error at block size of 25,000 was slightly higher than that of the previous settings, the overall estimation trend remained consistent, and no sharp decline in computational performance was observed. In contrast, the block size of 10,000 and 5,000 showed lower estimation accuracy than the other settings. These results indicate that overly small segmentation sizes disrupt the spatial continuity of features and exert a continuous effect on subsequent leaf area quantification.

**Table 3:** Results of leaves area error analysis.

Leaf	Unit (cm^2^) \ Indicator	Block size of 35,000	Block size of 30,000	Block size of 25,000	Block size of 20,000	Block size of 15,000	Block size of 10,000	Block size of 5,000
Area	MAE	9.12	8.98	10.19	9.27	9.09	11.53	12.42
	RMSE	25.83	25.66	26.94	26.22	25.71	27.81	35.13
	Accuracy	89.51%	89.67%	88.28%	89.34%	89.54%	86.74%	85.71%

To investigate the causes of the large error in leaf area estimation with block size of 5,000, we recall the *F*1 scores and IoU from the preceding phase. Compared to other block size settings, the main stem segmentation performance was the poorest with block size of 5,000 with substantial confusion between the predicted main stem and leaf pixels. This was a major factor contributing to the low leaf area calculation accuracy.

We also noticed that the calculated leaf area is generally larger than the actual measured values, indicating a systematically positive bias. Potential reasons are two folds. First, errors in the semantic segmentation process, particularly around the main stem region, lead to inaccurate delineation of leaf boundaries. As a result, portions of stem point clouds are incorrectly included in leaf point cloud sets, producing false leaf - stem connections. During leaf area computation, the surface fitting algorithm incorporates these stem points into the surface integration, which inflates the estimated leaf area. Besides, during surface fitting, a thin edge forms along the leaf perimeter, causing the calculated leaf area to exceed the actual area. The thickness of this thin edge is regulated by the Poisson fitting function, with optimal parameters varying across different leaf instances. However, this experiment used a uniform reconstruction depth of 8 for all leaves instances, contributing to the quantification error in leaf area measurements. Future work will build on this foundation by introducing dynamic thresholds that adaptively adjust surface-fitting hyperparameters according to leaf morphology, thereby mitigating these errors.

## 4 Conclusion

Leaves, as key plant organs, exhibit phenotypic characteristics that are vital indicators of plant growth, environmental adaptability, and photosynthetic efficiency. Consequently, leaf phenotyping analysis is fundamental in plant science research and holds significant practical value in agricultural productivity assessment, ecological monitoring, and genetic improvement of plant varieties. Three widely cultivated tomato cultivars (Merlice, Brioso, and Gardener Delight) were selected for this study. Deep-learning-based segmentation was employed to localize leaf targets within the point cloud. For the purpose of mitigating the information loss associated with the down sampling process required by conventional deep neural networks, the proposed work employed a distributed segmentation strategy, where the original point cloud was partitioned into multiple subsets in spatial order. Semantic segmentation was performed on each subset using PointNet++, and the final result was generated by merging the segmentation outputs from all subsets. To further segment leaf points into instances, the DBSCAN clustering algorithm was used based on the semantic segmentation result. Key phenotypic traits of leaves, including length, width, and area, were quantitatively characterized. Principal component analysis was used to generate the oriented bounding box of the target leaf, and leaf width was defined as the side length of the bounding box aligned with the second principal axis. Leaf area was quantified by fitting a surface to the leaf points using Poisson reconstruction. Leaf length was estimated using a geometric approximation to reduce errors induced by leaf curvature. Evaluation results showed that the distributed segmentation strategy achieved the best *F*1 scores of 0.98 with block size set to 30,000. The mean average errors of leaf length, leaf width, and leaf area estimations were 2.09cm, 1.78cm and 8.98cm^2^ respectively, with corresponding percentage errors of 91.98%, 92.66%, and 89.67% respectively.

Nevertheless, the proposed phenotyping pipeline for tomato plant leaves still maintains certain limitations. Although the distributed segmentation strategy presented in this paper improved segmentation performance by eliminating the need for conventional down sampling, contextual information was not effectively propagated across adjacent subsets. As a result, local structural information may become fragmented, leading to boundary artifacts in the merged segmentation results. Further improvements can be conducted by incorporating Long Short Term Memory (LSTM), Gated Recurrent Unit (GRU), and Bidirectional Recurrent Neural Network (Bi-RNN) to establish the communication mechanism across subsets. On the other hand, in complex scenarios with significant leaf interlacing, DBSCAN clustering may fail to segment leaf points into instances due to the absence of clear density gaps among instances. More robust instance segmentation approaches, for instance, deep-learning-based methods, will be considered in the future if the dataset is sufficiently large.

## Data Availability

The original contributions presented in the study are included in the article/supplementary material. Further inquiries can be directed to the corresponding author.

## Footnotes

1 The official website of Netherlands Plant Eco-phenotyping Centre (NPEC): https://www.npec.nl/

2 The dataset used in this study is available at: https://www.kaggle.com/datasets/xinbolai/vtc-tomato

3 The generation of 3D oriented bounding box can be achieved via function get_oriented_bounding_box() in Open3D library. The link for the function is presented here: https://www.open3d.org/docs/latest/python_api/open3d.geometry.AxisAlignedBoundingBox.html

4 It can be derived from Equation 3 that the relative approximation error of leaf length is smaller than 31.77% when the leaf curvature is smaller than 1/3.
